# Supplementary material for: MetaRibo-Seq measures translation in microbiomes
Source: Nat Commun. 2020 Jun 29;11:3268. doi: 10.1038/s41467-020-17081-z (PMC7324362; doi:10.1038/s41467-020-17081-z)
Supplement: Supplementary file 10 — Supplementary Data 7 [file 41467_2020_17081_MOESM10_ESM.zip › File2/Confidence_High_Taxonomy/86200_out.krona.html]

Javascript must be enabled to view this page.

members
magnitude
magnitudeUnassigned
count
unassigned
taxon
rank

86200\_out

6

superkingdom
2
6

phylum
1239
6

6
186801
class

6
order
186802

family
186803
6

33042
genus
6

species
33043

SRS014855\_contig\_number\_1573SRS014923\_contig\_number\_13751SRS015854\_contig\_number\_19676SRS050925\_contig\_number\_25413SRS144714\_contig\_number\_29252
5


SRS098571\_contig\_number\_59945
1
1263071
species
